# 3D Simulation-Driven Design of a Microfluidic Immunosensor for Real-Time Monitoring of Sweat Biomarkers

**DOI:** 10.3390/mi15080936

**Published:** 2024-07-23

**Authors:** Nessrine Jebari, Elisabeth Dufour-Gergam, Mehdi Ammar

**Affiliations:** Center for Nanosciences and Nanotechnology (C2N), CNRS UMR 9001, University of Paris-Saclay, 91120 Palaiseau, France

**Keywords:** microfluidic immunosensor, 3D simulation, capacitive sensing, magnetofluidic manipulation, real-time monitoring

## Abstract

This study presents the design and comprehensive 3D multiphysics simulation of a novel microfluidic immunosensor for non-invasive, real-time detection of pro-inflammatory biomarkers in human sweat. The patch-like device integrates magnetofluidic manipulation of antibody-functionalized magnetic nanoparticles (MNPs) with direct-field capacitive sensing (DF-CS). This unique combination enhances sensitivity, reduces parasitic capacitance, and enables a more compact design compared to traditional fringing-field approaches. A comprehensive 3D multiphysics simulation of the device, performed using COMSOL Multiphysics, demonstrates its operating principle by analyzing the sensor’s response to changes in the dielectric properties of the medium due to the presence of magnetic nanoparticles. The simulation reveals a sensitivity of 42.48% at 85% MNP occupancy within the detection zone, highlighting the sensor’s ability to detect variations in MNP concentration, and thus indirectly infer biomarker levels, with high precision. This innovative integration of magnetofluidic manipulation and DF-CS offers a promising new paradigm for continuous, non-invasive health monitoring, with potential applications in point-of-care diagnostics, personalized medicine, and preventive healthcare.

## 1. Introduction

The global rise of chronic diseases, particularly those linked to inflammation, underscores the critical need for accessible and reliable health monitoring tools [1,2]. Early detection of pro-inflammatory states can enable timely interventions, potentially mitigating disease progression and improving patient outcomes [3,4,5]. However, traditional diagnostic methods are often invasive, time-consuming, and costly, limiting their utility for continuous, real-time health assessments [6,7,8]. This has fueled the development of wearable biosensors capable of non-invasively monitoring physiological biomarkers in biofluids like sweat [9,10,11].

Microfluidic platforms have emerged as a promising avenue for developing such wearable biosensors, offering unique advantages in terms of miniaturization, automation, and integration of multiple functionalities [12,13,14]. These platforms enable precise manipulation of minute fluid volumes, reducing reagent consumption and waste generation, and facilitating rapid analysis [15,16,17]. The integration of microfluidics with diverse sensing modalities, such as magnetic and capacitive sensing, has opened up new possibilities for creating sophisticated, yet compact, biosensors capable of multiplexed detection and real-time monitoring [18,19,20].

Among various biosensing modalities, magnetic and capacitive sensing have garnered significant attention due to their inherent sensitivity and compatibility with miniaturized, wearable platforms [21,22,23]. Magnetic biosensors, leveraging the unique properties of magnetic nanoparticles, have demonstrated exceptional potential for detecting a wide range of analytes, including proteins, nucleic acids, and even whole cells [24,25,26,27]. Capacitive biosensors offer label-free detection with high sensitivity and rapid response times, making them well-suited for real-time monitoring applications [28,29,30].

However, current magnetic and capacitive biosensors face challenges in their translation to continuous, wearable health monitoring. Many magnetic biosensors rely on complex signal processing and external instrumentation, hindering their integration into user-friendly wearable devices [31]. Moreover, the reliance on indirect detection methods may limit their sensitivity to low-abundance biomarkers. Capacitive biosensors, while often simpler in design, can be susceptible to non-specific binding and interference from other sweat components, potentially compromising accuracy and reliability [32]. Further, the need for specific surface functionalization in many capacitive sensors limits their versatility and reusability.

To overcome these limitations, we propose a novel dual-unit microfluidic biosensor that combines the strengths of both magnetofluidic manipulation and direct-field capacitive sensing (DF-CS) in a single, integrated platform. By leveraging magnetic pre-concentration to enhance sensitivity and a streamlined direct-field capacitive sensing (DF-CS) mechanism for direct quantification, the biosensor enables continuous, real-time monitoring of pro-inflammatory biomarkers in human sweat. This innovative combination of magnetofluidic manipulation and capacitive sensing brings a significant advantage: enhanced specificity without the need for complex surface functionalization. Our approach circumvents the issues associated with surface modifications, allowing the sensor to be easily adapted for detecting a wide array of biomarkers, enhancing its reusability and reducing the risk of non-specific binding. Notably, the use of DF-CS addresses the shortcomings of the more common fringing-field capacitive sensing (FF-CS) method, which is particularly sensitive to surface chemistry and prone to interference from other sweat components. In contrast, DF-CS, with its direct measurement within the sensing region, minimizes the impact of surface conditions, maximizes the interaction between the electric field and the biomarker-bound magnetic nanoparticles and offers improved sensitivity, reduced parasitic capacitance, and a more compact design—all crucial for wearable applications. This unique combination of technologies, coupled with the potential for scalable fabrication, holds promise for realizing a truly wearable, non-invasive biosensor for continuous health monitoring.

In this paper, we present the design and comprehensive 3D multiphysics simulation of this proposed novel microfluidic immunosensor for real-time, non-invasive detection of pro-inflammatory biomarkers in human sweat. Our study focuses on the theoretical modeling and simulation of the biosensor’s performance, providing a comprehensive understanding of its underlying principles and potential efficacy. Experimental validation of the biosensor will be the subject of future work.

## 2. Overview

### 2.1. Problem Formulation

**Figure 1:**
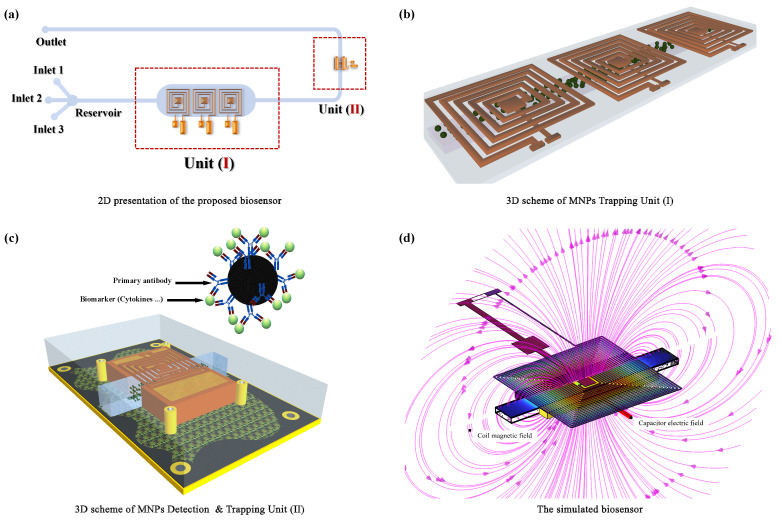
**Key components of the proposed microfluidic immunosensor.** (**a**) Overview of the sensor design, showcasing the primary and secondary units. (**b**) 3D layout of the MNPs Trapping Unit (I), featuring a serial coil configuration. (**c**) 3D schematic of the MNPs Detection and Trapping Unit (II), detailing the use of functionalized MNPs. (**d**) COMSOL Multiphysics-generated 3D model demonstrating the operational dynamics of the biosensor.

The proposed biosensor, illustrated in Figure 1, represents a novel approach to continuous, real-time monitoring of pro-inflammatory biomarkers in human sweat. This non-invasive wearable patch is engineered to provide valuable insights into an individual’s physiological state and enable early detection of inflammation-related conditions. To achieve this, the biosensor employs a dual-unit structure designed to optimize both the efficiency and accuracy of biomarker detection.

#### 2.1.1. Unit I: Immunocomplex Formation and Pre-Concentration

Unit I (Figure 1b) serves as the initial sample preparation stage, facilitating the specific capture and concentration of target biomarkers. The unit incorporates an array of planar microcoils that generate a localized magnetic field gradient within the microfluidic channel. This magnetic field gradient simultaneously attracts and immobilizes antibody-functionalized magnetic nanoparticles (MNPs) and promotes active mixing within the channel, ensuring a homogeneous distribution of both biomarkers and MNPs. This dual functionality enhances the biosensor’s sensitivity by increasing the probability of MNP-biomarker interactions, leading to rapid and efficient formation of immunocomplexes. By accelerating the kinetics of immunocomplex formation and concentrating the target biomarkers, Unit I plays a crucial role in expediting the overall assay speed and enhancing sensitivity, enabling faster detection of even low-abundance biomarkers in complex biological samples like sweat.

#### 2.1.2. Unit II: Selective Biomarker Detection and Quantification via Capacitive Sensing

Unit II (Figure 1c) serves as the selective detection zone of the biosensor, employing a direct-field capacitive sensing (DF-CS) mechanism to quantify biomarker concentrations. A microcoil positioned above the channel captures the biomarker-tagged MNPs from Unit I and directs them into a microfluidic channel embedded within a dielectric domain defined by dual parallel electrodes. This configuration creates a capacitive structure, where the presence of MNPs within the electric field alters the dielectric properties of the medium, leading to a measurable shift in capacitance. This capacitance change is directly proportional to the concentration of biomarker-tagged MNPs, providing an indirect measure of the biomarker concentration in the original sample. By precisely measuring this capacitance shift using integrated electronics, the biosensor can accurately quantify the levels of pro-inflammatory biomarkers in sweat. By maximizing the interaction between the electric field and the MNP-biomarker complexes, the electrodes ensure a high signal-to-noise ratio, enabling the reliable detection of even minute quantities of biomarkers. This enhanced sensitivity is particularly important for early disease detection and monitoring, where low levels of biomarkers may be present.

The compartmentalized design, featuring distinct units for immunocomplex formation and capacitive detection, confers significant advantages over conventional single-unit biosensors. This spatial separation of functions enables precise control over assay conditions, ensuring optimal performance at each stage. The isolation of the capture and detection zones minimizes cross-contamination and non-specific interactions, enhancing the accuracy and reliability of biomarker quantification. Furthermore, this modular architecture facilitates independent optimization of each unit, allowing for greater flexibility in tailoring the biosensor to specific applications. As a result, this innovative design is particularly well-suited for the continuous, real-time monitoring of pro-inflammatory biomarkers in wearable applications, where robustness, accuracy, and user-friendliness are paramount.

To rigorously evaluate and optimize the design of our proposed biosensor, we developed a comprehensive 3D multiphysics model (Figure 1d). This model integrates three distinct domains, each representing a critical physical process contributing to the sensor’s functionality:

1. **Electromagnetic Domain:** This domain focuses on simulating the magnetic field distribution generated by the microcoil array. By solving Maxwell’s equations within this domain, we can accurately predict the magnetic forces acting on the functionalized MNPs, enabling us to optimize the coil design for efficient MNP trapping and concentration.
2. **Fluid Dynamics Domain:** This domain simulates the behavior of the biofluid (sweat) within the microfluidic channels, considering the complex interactions between the fluid, MNPs, and channel walls. This simulation helps optimize channel geometry and flow conditions for efficient biomarker capture.
3. **Electrostatic Domain:** The electrostatic domain models the electric field distribution between the electrodes and the capacitive response resulting from the presence of MNPs in the detection zone. Precise modeling of the electrical properties ensures accurate biomarker quantification.

By utilizing the finite element method (FEM) in COMSOL Multiphysics v6.0, we numerically solve the governing equations for the magnetic, fluidic, and electric fields within their respective domains. This comprehensive multiphysics approach provides valuable insights into the biosensor’s operational dynamics, facilitating iterative design refinement. To ensure the accuracy and reliability of our model, we validated the simulation results through convergence studies with refined mesh sizes and time steps.

### 2.2. Physics and Mathematical Framework of the Biosensor

**Figure 2:**
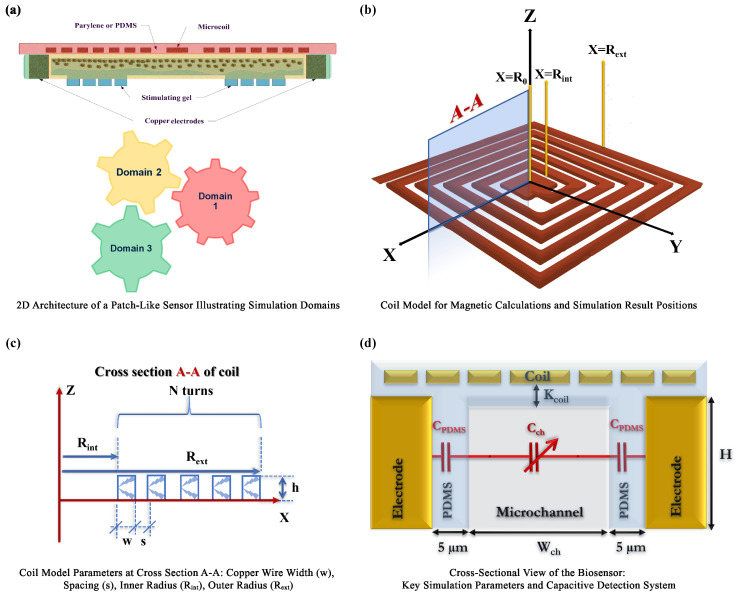
**Simulation model of a patch-like sensor.** (**a**) 2D architecture illustrating simulation domains. (**b**) Detailed coil model used for magnetic field calculations, indicating specific positions utilized in simulations. (**c**) Coil parameters at cross section A-A, detailing copper wire width (w), spacing (s), inner radius ($R_{\mathrm{int}}$), and outer radius ($R_{\mathrm{ext}}$). (**d**) Cross-sectional view that highlights key simulation parameters and the capacitive detection system.

Building upon the introduction and design overview, this section delves into the fundamental physical and mathematical principles governing the operation of the microfluidic immunosensor. Through a detailed analysis of the three critical domains illustrated in Figure 2a, we aim to provide a rigorous and comprehensive understanding of the sensor’s functional mechanisms.

#### 2.2.1. Domain 1: Planar Coils

Domain 1 focuses on characterizing the magnetic field generated by the coils within the biosensor. To optimize magnetic field strength while minimizing power consumption, we evaluated four distinct copper planar coil designs (Figure 2b,c), each varying in outer radius and turn count (R500 to R2000). All designs maintained consistent copper wire dimensions (width (w) = 10 μm, height (h) = 10 μm, and a separation between adjacent wires (s) = 10 μm) to ensure a fair comparison.

Maxwell’s equations were employed to model the magnetic field distribution around the coils, utilizing the magnetic vector potential (*A*) formulation: [33]

(1) B=∇×A

Additionally, the Ampere-Maxwell equation, incorporating current density (*J*), was used: [33]

(2) ∇×H=J

The relationship between magnetic flux density (*B*), magnetic field strength (*H*), and the medium’s relative permeability ($\mu_{r}$) is given by: [33]

(3) $$B=\mu_{0}\mu_{r}H$$

where $\mu_{0}=4\pi \times 10^{-7}\;N/A^{2}$ is the permeability of free space. Quadratic discretization in the finite element method (FEM) ensured accurate simulation of electromagnetic fields.

Coil efficiency was quantified using the power merit factor ($M_{p}$), defined as the ratio of maximum magnetic field strength ($B_{\max}$) to power consumption (P = U × I). The objective was to identify coil dimensions and operational parameters that optimize the balance between field intensity and power consumption for long-term device performance.

#### 2.2.2. Domain 2: Microfluidic Platform

Domain 2 simulates the microfluidic platform, focusing on the dynamics of sweat flow and magnetic nanoparticle (MNP) transport. Sweat, primarily composed of water, is modeled as an incompressible fluid using the Navier-Stokes equations (Equation (4)) [34]:

(4) $$\rho \frac{\partial u}{\partial t}+\rho \left(u\cdot \nabla \right)u=-\nabla P+\eta \nabla^{2}u+F$$

MNPs within the sweat flow experience both magnetofluidic forces (Equation (5)) due to interaction with the magnetic field gradient, and viscous drag forces (Equation (6)) modeled using Stokes’ law [35,36]:

(5) $$F_{\mathrm{map}}=\frac{1}{4}\pi \mu_{r}\mu_{0}D^{3}\left[\frac{\mu_{r,p}-\mu_{r}}{\mu_{r,p}+2\mu_{r}}\nabla H^{2}\right]$$

(6) $$F_{D}=6\pi \eta r\left(u-v_{p}\right)$$

where *D* is the particle diameter, $\mu_{r,p}$ is the relative permeability of the particle, $v_{p}$ is the particle velocity, η is the fluid’s viscosity, *r* is the particle radius, and u is the fluid velocity.

To solve these complex equations governing particle motion, the Generalized alpha method (GAM) is employed, ensuring accurate temporal evolution and suppressing spurious oscillations for simulation stability.

#### 2.2.3. Domain 3: Capacitive Electrodes

Domain 3 focuses on modeling the capacitive sensing mechanism that underlies the biosensor’s detection capabilities. As shown in Figure 2d, strategically placed metallic electrodes within the microfluidic channel form a capacitor, highly sensitive to changes in capacitance induced by the presence of MNPs. This direct-field capacitive sensing (DF-CS) approach offers significant advantages in sensitivity and label-free detection.

The capacitance (*C*) of a parallel plate capacitor is described by:

(7) $$C=\frac{\epsilon_{0}\epsilon_{r}A}{d}$$

where $\epsilon_{0}$ represents the vacuum permittivity, $\epsilon_{r}$ is the relative permittivity of the medium, *A* denotes the electrode area, and *d* is the separation distance [37].

When biomarker-tagged MNPs accumulate in the detection zone, they alter the effective relative permittivity ($\epsilon_{r}$), leading to a measurable change in capacitance (ΔC):

(8) $$\Delta C=\frac{\epsilon_{0}A}{d}\left(\epsilon_{r,\mathrm{after}}-\epsilon_{r,\mathrm{before}}\right)$$

Here, $\epsilon_{r,\mathrm{before}}$ and $\epsilon_{r,\mathrm{after}}$ represent the relative permittivity before and after MNP introduction, respectively. This change is directly proportional to the MNP concentration, enabling biomarker quantification.

To accurately model the sensor’s response, we consider the microfluidic channel’s structure and estimate the equivalent capacitance ($C_{eq}$), accounting for the PDMS walls and the variable channel capacitance:

(9) $$C_{eq}=\frac{\epsilon_{0}\epsilon_{r,\mathrm{PDMS}}\epsilon_{r,\mathrm{ch}}}{\epsilon_{r,\mathrm{PDMS}}d_{\mathrm{ch}}+2\epsilon_{r,\mathrm{ch}}d_{\mathrm{PDMS}}}A$$

where $\epsilon_{r,\mathrm{PDMS}}$ and $\epsilon_{r,\mathrm{ch}}$ represent the dielectric constants of PDMS and the channel, respectively (Figure 2d). This comprehensive model, integrating DF-CS and magnetic trapping, provides a robust framework for understanding and optimizing the detection and quantification of MNP-tagged biomarkers.

In practice, the measured capacitance change (ΔC) is used to determine biomarker concentration by referencing a pre-established calibration curve, which relates ΔC to known biomarker concentrations. This approach enables precise and reliable determination of biomarker levels in real-world samples.

### 2.3. Implementation Details

Given the intricate interplay of electromagnetic, fluidic, and nanoparticle dynamics within the biosensor, a robust multiphysics simulation strategy was essential. COMSOL Multiphysics, with its versatile capabilities, was chosen as the simulation platform due to its ability to seamlessly integrate diverse physical models. The simulation process encompassed three primary modules:

- **AC/DC Module for Electromagnetic Field Simulation:** This module was employed to rigorously solve Maxwell’s equations and accurately characterize the magnetic field distribution generated by the microcoil array and the electric field established between the electrodes. Accurate simulation of these fields is fundamental to understanding their influence on the behavior of the biological media and the MNPs, ultimately determining the efficacy of biomarker detection.
- **CFD Module for Fluid Dynamics Analysis:** To model the intricate flow of biofluids, specifically sweat, through the microchannels, we utilized the Computational Fluid Dynamics (CFD) Module. By solving the Navier-Stokes equations, we could accurately capture fluid behavior, including velocity profiles, pressure gradients, and shear stresses. This comprehensive understanding of fluid dynamics is essential for optimizing microchannel geometry and flow conditions to ensure efficient transport and interaction of biomarkers with the MNPs.
- **Particle Tracing Module for Nanoparticle Dynamics Investigation:** The Particle Tracing Module enabled the simulation of the dynamic behavior of magnetic nanoparticles within the microfluidic environment. By incorporating the forces acting on the MNPs, including magnetic forces and viscous drag, we could predict their trajectories and interactions with the surrounding fluid and channel walls. This analysis provided critical insights into the mechanisms governing MNP capture, concentration, and ultimately, biomarker detection.

The accuracy and efficacy of the simulations were contingent upon the meticulous selection of design parameters and material constants (Table 1 and Table 2). These parameters, derived from experimental data, literature review, and preliminary simulations, encompassed variables such as coil current, electrode height, nanoparticle diameter, channel dimensions, and material properties. This rigorous approach ensured that our models accurately reflected the real-world behavior of the biosensor, facilitating informed design decisions and performance optimization.

## 3. Results and Discussion

### 3.1. Optimizing Coil Configurations: Balancing Efficiency and Functionality

Microfluidic biosensors frequently utilize magnetic fields for the controlled manipulation of magnetic nanoparticles (MNPs) to perform various functions, such as analyte capture, concentration, and detection. Integrated planar microcoils, compared to traditional permanent magnets, offer superior adaptability for biosensing applications due to their dynamic control over magnetic field strength and configuration through modulation of input current. This enables precise spatiotemporal manipulation of MNPs within microfluidic channels, facilitating targeted biochemical interactions and efficient bioassay operation. Furthermore, the ability to deactivate microcoils, eliminating residual magnetism, prevents unintended MNP aggregation or interference, ensuring assay reproducibility and reliability. Building upon our prior research on coil design [38,39,40,41], this study investigates coil configurations optimized for both MNP trapping and detection within the biosensor, aiming to create broader trapping zones and more uniform magnetic fields for enhanced magnetofluidic performance.

#### 3.1.1. Magnetic Field Analysis

**Figure 3:**
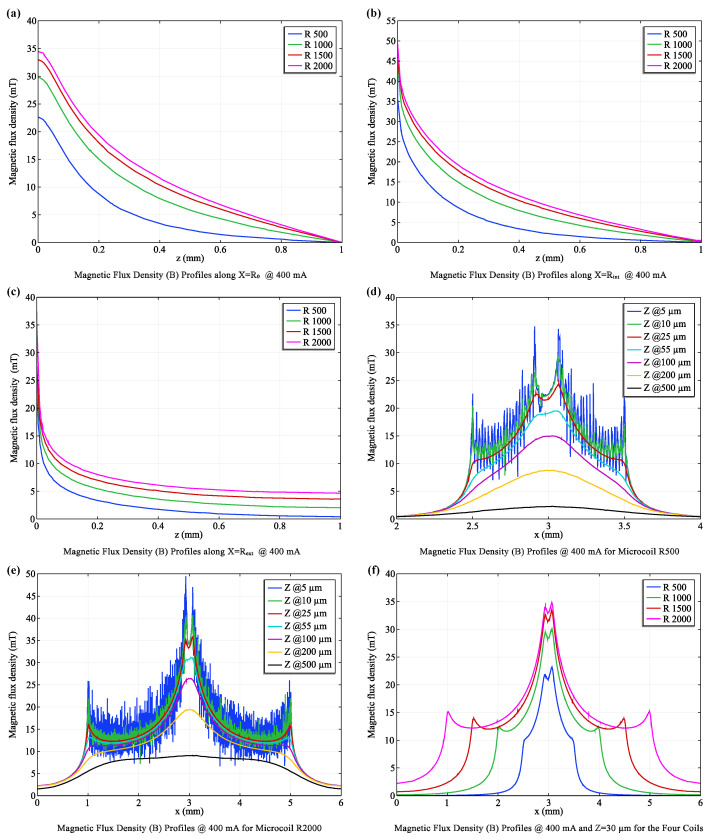
**Magnetic flux density profiles across paths and coils.** (**a**) Magnetic field profile along $R_{0}$ for four coils, emphasizing the central field strength. (**b**) Field characteristics internal to the coils along $R_{\mathrm{int}}$. (**c**) Peripheral field behavior along $R_{\mathrm{ext}}$, illustrating field distribution at the coil edges. (**d**) Magnetic flux density (*B*)-profile for the R500 coil, and (**e**) for the R2000 coil, measured across seven distinct z-paths. (**f**) Combined profiles at Z = 30 μm, determining the optimal *Z*-position for maximizing flux density while minimizing field ripple.

We rigorously analyzed four single-coil configurations (R500 to R2000) with a constant current of 400 mA, assessing magnetic flux density (B) in both vertical ($B_{z}$) and horizontal ($B_{x}$) directions at various distances along the microfluidic channel (Figure 3). This allowed us to characterize the strength, uniformity, and directionality of the generated magnetic field for each coil design.

Our findings are consistent with the Biot-Savart law, which describes the inverse relationship between magnetic field strength and distance from a current-carrying conductor. As illustrated in Figure 3a–c, the magnetic flux density exhibited a decay that closely followed a $\frac{1}{x}$ curve approximation as the distance from the coil surface increased. The R2000 coil, with its greater number of turns, consistently generated stronger magnetic fields compared to the R500 coil at all evaluated positions (X = $R_{0}$, $R_{\mathrm{int}}$, $R_{\mathrm{ext}}$). This observation aligns with the theoretical expectation that magnetic field strength increases linearly with the number of turns in a coil for a given current.

To quantify these observations, the magnetic flux density was assessed at three key positions relative to the coils (Figure 3d,e):

- **X = $R_{0}$ (Coil Center)**: The R2000 coil (97 turns) exhibited a substantially stronger vertical magnetic flux density ($B_{z}$) of 20.58 mT compared to the R500 coil (10.05 mT), highlighting the direct influence of coil turns on field strength.
- **X = $R_{int}$ (Intermediate Distance)**: This trend persisted at an intermediate distance within the coil, with the R2000 coil maintaining a higher average $B_{z}$ (21.92 mT) than the R500 coil (11.58 mT).
- **X = $R_{ext}$ (Coil Edge)**: Even at the coil’s outer edge, the R2000 coil produced a stronger $B_{z}$ (9.42 mT) compared to the R500 coil (5.19 mT).

Importantly, the simulations revealed a higher degree of magnetic flux density uniformity in closer proximity to the coil surface (X ≈ $R_{0}$). This finding is of paramount importance for MNP trapping applications, as it ensures a consistent and robust magnetic force acting on the MNPs within the trapping zone, facilitating their efficient capture and concentration. As expected from electromagnetic principles, the magnetic flux density exhibited a clear trend of decreasing with increasing distance from the coil surface. However, the larger R2000 coil, due to its greater number of turns, consistently generated higher flux densities compared to the smaller coils. This translates to a broader and more effective trapping zone, making the R2000 coil the optimal choice for the MNP Trapping Unit (Unit I) where efficient and reliable MNP capture is crucial for subsequent biomarker detection.

#### 3.1.2. Optimal Field Uniformity

While achieving a strong magnetic field is essential for MNP manipulation, ensuring its uniformity is equally critical for consistent and predictable particle behavior. Our analysis reveals that the magnetic field generated by the microcoil array exhibits the highest degree of uniformity at a distance of approximately 30 µm from the coil surface (Figure 3d–f). This optimal distance can be attributed to the “rippling effect”, a phenomenon arising from the close proximity of coil turns. This effect leads to minor variations in current density, resulting in slight fluctuations in the magnetic field intensity near the coil surface.

To mitigate the rippling effect and ensure a uniform magnetic field within the trapping zone, we incorporate a spacer layer, typically implemented through the microfluidic channel design, between the coil and the fluidic medium. This strategic placement ensures that the MNPs experience a consistent magnetic force, minimizing unwanted torque or rotational forces that could disrupt their alignment and movement.

#### 3.1.3. Power Efficiency Considerations

**Figure 4:**
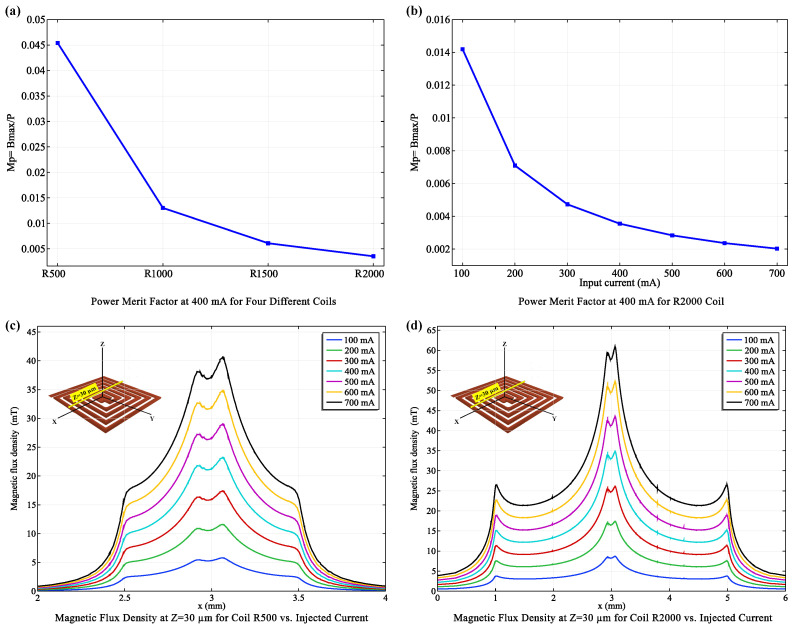
**Analysis of Power Merit Factors and Magnetic Flux Density based on Injected Current.** (**a**) Power merit Factor comparison for four coils at 400 mA, highlighting performance differences. (**b**) Power merit Factor evaluation for the R2000 coil across various current values to assess efficiency. (**c**) Magnetic flux density profiles for the R500 coil at different current levels. (**d**) Magnetic flux density profiles for the R2000 coil at various currents, correlated with the power merit factors discussed above.

The R2000 coil, while producing a stronger magnetic field, exhibited lower power efficiency compared to the R500 coil, as indicated by its inferior power merit factor ($M_{p}$) (Figure 4a). $M_{p}$, defined as the ratio of maximum magnetic flux density to power consumption, is a critical parameter for evaluating coil performance, especially in wearable biosensors where energy conservation is paramount. The R500 coil, with its higher Mp, was selected for the MNP Detection Unit (Unit II), as it provides sufficient field strength for MNP capture while minimizing power consumption, thereby extending battery life.

Our analysis further revealed an inverse relationship between the R2000 coil’s power efficiency and the applied current (Figure 4b,d). While higher currents increased the magnetic field strength, they also led to a disproportionate rise in power consumption. For example, $M_{p}$ values for the R2000 coil increased substantially when currents were raised from 100 mA to 400 mA, but a sharp decline in efficiency was observed at 700 mA.

This trade-off between field strength and power efficiency necessitates careful optimization. Based on our findings, an optimal operational current range of 100–400 mA was identified for the R2000 coil in the MNP Trapping Unit (Unit I). This range balances the need for sufficient magnetic field strength for effective MNP trapping with the goal of minimizing power consumption for sustainable device operation.

#### 3.1.4. Tailoring Coils to Specific Functions

**Figure 5:**
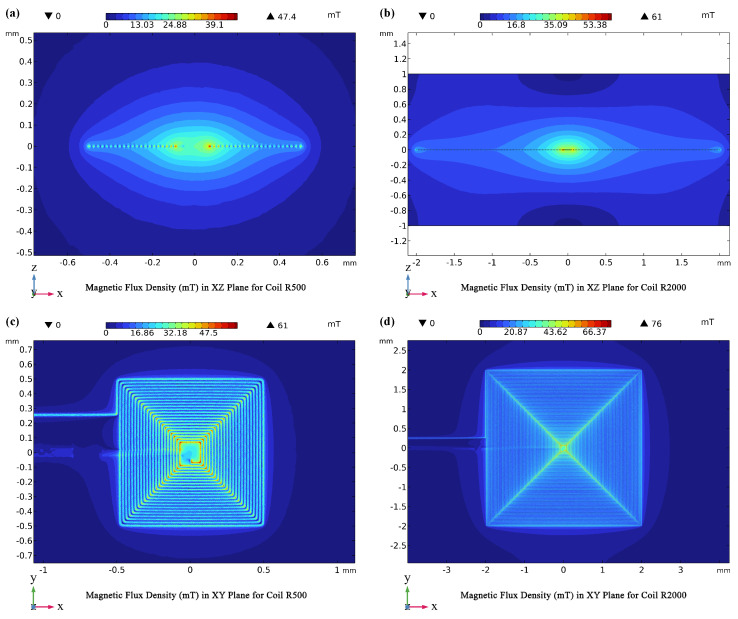
**Magnetic flux density maps across different planes and coils.** (**a**) Magnetic flux density in the xz-plane for the R500 coil, illustrating field distribution. (**b**) xz-plane map for the R2000 coil, showing detailed *B*-field gradients. (**c**) Magnetic flux density in the xy-plane for the R500 coil, depicting field uniformity. (**d**) xy-plane map for the R2000 coil, highlighting enhanced magnetic density concentration at both corners and the center.

Our findings highlight the importance of tailoring coil design to specific biosensor functions. The R2000 coil, with its greater number of turns, generates a 20% stronger magnetic flux density than the R500 coil at 400 mA (Figure 5). This results in a significantly wider trapping zone, critical for efficient MNP capture and concentration in the MNP Trapping Unit (Unit I). Both coils exhibit high flux densities in their central region.

Thus, the R2000 coil, with its strong and broad magnetic field, is ideally suited for Unit I, where enhanced field strength maximizes MNP capture. Conversely, the R500 coil, with its superior power efficiency (Figure 4a), is better suited for the MNP Detection Unit (Unit II), where a smaller field suffices, and energy conservation is prioritized to extend battery life.

Strategically selecting and optimizing coil configurations based on their roles within the biosensor significantly enhances overall performance, sensitivity, and energy efficiency. This tailored approach not only improves current capabilities but also informs future wearable health monitoring technologies.

In the following section, we delve into simulation results concerning fluid dynamics and magnetic trapping efficiency, further elucidating how coil geometry and flow dynamics influence MNP trapping and detection.

### 3.2. Fluid Dynamics and Magnetic Trapping Performance in Microfluidic Platform

We employ computational fluid dynamics (CFD) simulations and magnetic field modeling to explore the interaction between coil configurations and fluid flow in microfluidic environments. Understanding these dynamics is vital for optimizing the trapping of magnetic nanoparticles (MNPs), crucial for enhancing biosensor functionality.

#### 3.2.1. Optimizing Microchannel Alignment

**Figure 6:**
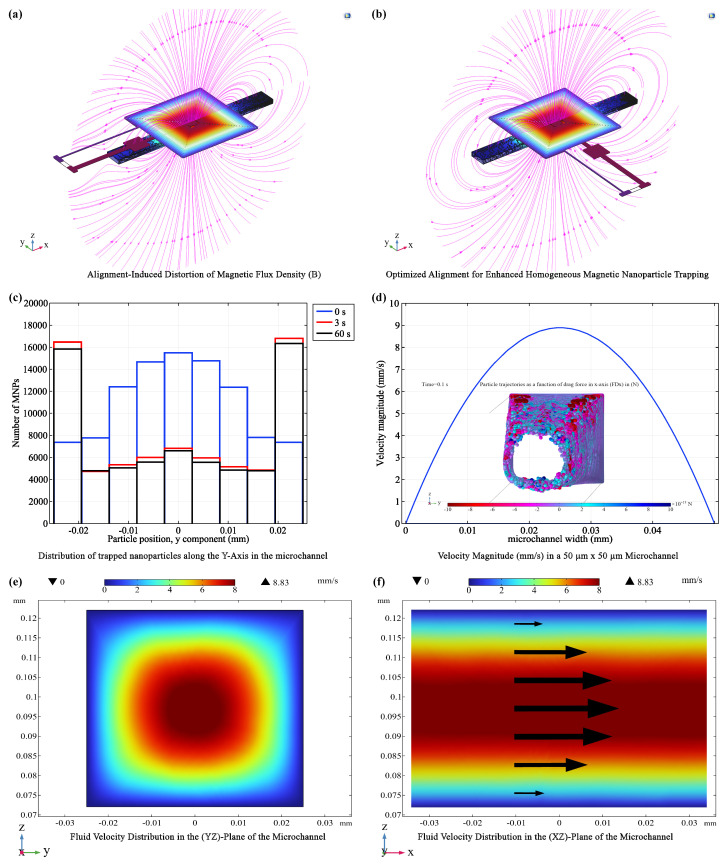
**Impact of Microchannel Configurations on Fluid Dynamics and MNPs Trapping.** (**a**) Asymmetric magnetic field within the microchannel and its influence on MNPs trapping. (**b**) Optimization of alignment for enhanced homogeneous MNPs trapping, achieving near-symmetric fields. (**c**) Spatial distribution of trapped MNPs along the *Y*-axis within the microchannel, captured at three distinct time points. (**d**) Fluid velocity profile across the channel height; inset shows MNPs positions at *t* = 0.1 s alongside the drag force expression, illustrating particle deceleration. (**e**) Fluid velocity map in the YZ-plane. (**f**) Fluid velocity map in the XZ-plane, highlighting regions of highest velocity.

The placement of the microchannel relative to the coil significantly impacts MNP trapping efficiency (Figure 6). Simulations revealed that a channel aligned parallel to the coil vias (Figure 6a) results in a non-uniform magnetic field distribution across the channel width. This non-uniformity leads to heterogeneous magnetic forces acting on the MNPs, causing uneven trapping and potentially reducing the overall capture efficiency. In contrast, aligning the microchannel perpendicular to the vias (Figure 6b) creates a more uniform magnetic field across the channel width. This uniformity significantly improves the homogeneity of MNP trapping, as evidenced by the more even distribution of MNPs observed in the simulations (Figure 6c). This finding underscores the importance of strategic microchannel placement to maximize the effectiveness of magnetic trapping and ensure consistent MNP behavior within the biosensor.

#### 3.2.2. Fluid Dynamics and MNP Trapping

In the optimized 50×50μm microchannel, laminar flow with a parabolic velocity profile is established (Figure 6d–f). This laminar flow regime, characterized by smooth, parallel layers of fluid with minimal mixing, is ideal for precise control and manipulation of MNPs within the microfluidic environment. MNPs flowing through the channel are subject to two competing forces: a magnetofluidic force, which pulls them towards regions of higher magnetic field strength (typically near the coil center), and a drag force, which opposes their movement and is proportional to the fluid velocity. Due to the parabolic velocity profile, the drag force is strongest at the center of the channel and weakens towards the walls. Conversely, the magnetic field gradient is steeper near the walls, resulting in an increased magnetofluidic force in these regions. The interplay between these forces dictates the MNP trajectories. As MNPs flow through the channel, they experience a gradual increase in the magnetofluidic force and a decrease in the drag force as they approach the channel walls. This results in a net force directing the MNPs towards the walls, where they accumulate due to the minimal flow velocity, facilitating their efficient capture (Figure 6d inset).

#### 3.2.3. Coil Performance in Trapping Efficiency

The trapping performance of each coil configuration (R500 to R2000) was systematically evaluated based on the optimized parameters from the previous section. The R2000 coil, with its larger size and stronger magnetic field, achieved the highest mean trapping efficiency (82.83%) and the lowest standard deviation (2.20%). The R1500, R1000, and R500 coils followed with efficiencies of 72.20%, 67.35%, and 60.18%, respectively. All coils reached a steady-state trapping regime rapidly (within 1 to 10 seconds), with the R2000 coil achieving this in just 1 s. This rapid trapping time directly influences the overall response time of the biosensor, as the subsequent detection of biomarkers through capacitive sensing can only occur after the MNPs have been effectively concentrated in the detection zone.

**Figure 7:**
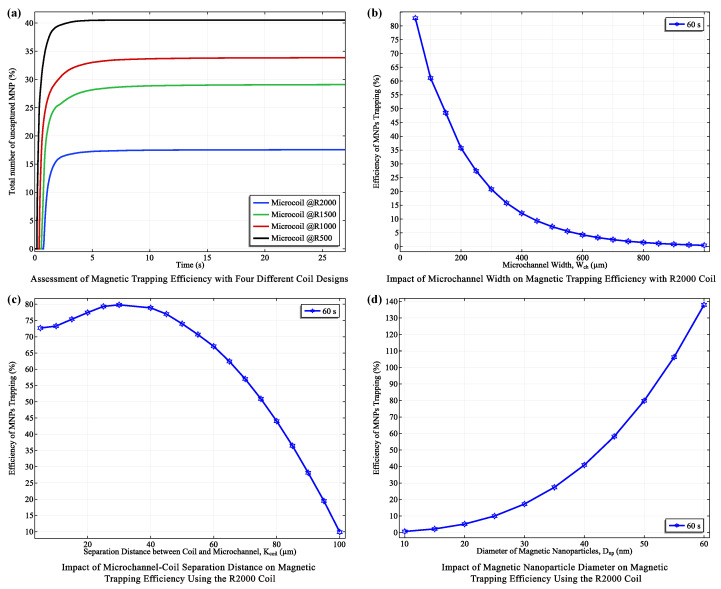
**Analysis of Magnetic Trapping Efficiency Under Varied Conditions.** (**a**) Comparative efficiency across four different coils, highlighting performance disparities. (**b**) Variation in magnetic trapping efficiency of the R2000 coil across different microchannel widths, measured at 60 s. (**c**) Efficiency trends at varying distances between the microchannel and the R2000 coil, observed at 60 s. (**d**) Dependency of magnetic trapping efficiency on the diameter of magnetic nanoparticles using the R2000 coil, assessed at 60 s.

To further optimize MNP trapping, we investigated the impact of microchannel width and coil-to-channel distance using the R2000 coil configuration. A strong negative correlation was found between microchannel width (ranging from 50μm to 1000μm) and trapping efficiency. Efficiency dropped precipitously from 82.83% at 50μm to 0.44% at 1000μm (Figure 7b), with channel width accounting for 73.3% of the variance in trapping efficiency. This highlights the crucial role of channel geometry in magnetic trapping systems. Narrower channels concentrate the magnetic flux, resulting in higher flux density and steeper field gradients within the channel, thereby enhancing MNP capture and retention.

The enhanced trapping efficiency observed in narrower microchannels is attributable to the magnetic field characteristics of the planar coil. Narrower channels concentrate magnetic flux, resulting in higher flux densities within the channel (Figure 6b). This, in turn, produces steeper magnetic field gradients (i.e., a more rapid change in field strength with distance), which exert stronger forces on MNPs, facilitating their capture and retention.

Furthermore, we investigated the impact of microchannel-coil separation distance (K_coil_) on trapping efficiency using the R2000 coil (Figure 7c). The highest efficiency (82.83%) occurred at K_coil_ = 30 μm, consistent with the optimal distance predicted based on magnetic field profiles where the “rippling effect” (fluctuations in field intensity due to the close proximity of coil turns) is minimized. Trapping efficiency decreased significantly as the separation exceeded 30 μm due to the weakening magnetic field.

Finally, we investigated the influence of nanoparticle diameter (D_NP_) on trapping efficiency using the R2000 coil at 400 mA (Figure 7d). Consistent with theoretical predictions, larger MNPs exhibited higher trapping efficiencies. This is attributed to the magnetofluidic force scaling with ($D_{\mathrm{NP}}^{3}$), while the drag force scales inversely with ($D_{\mathrm{NP}}^{2}$). Consequently, larger MNPs experience a greater magnetic force relative to drag, facilitating their capture and retention.

Based on the comprehensive optimization process detailed above, we have identified the following parameters as ideal for maximizing magnetic trapping efficiency in the biosensor: a microchannel width of 50μm, a coil-to-channel separation distance of (K_coil_) 30μm, and a MNP diameter (D_NP_) of 50nm. These conditions not only ensure optimal MNP capture and retention but also promote a high degree of magnetic field uniformity, crucial for consistent MNP behavior within the trapping zone. With these parameters established, the subsequent focus of our study shifts to the innovative capacitive detection system, which will leverage the efficient MNP trapping capabilities to achieve high sensitivity and specificity in biomarker detection.

### 3.3. Capacitive Sensing Performance and Implications

Following optimization of the coil configurations and fluid dynamics for MNP trapping, we integrated capacitive sensing technology into the biosensor to enhance biomarker monitoring sensitivity. This approach synergistically combines magnetic trapping with direct-field capacitive detection of biomarker-bound MNPs. Based on our prior analysis, the R500 coil, exhibiting superior power efficiency and sufficient trapping capacity, was selected for the combined Trapping & Detection unit (Unit II).

#### 3.3.1. Electrode Material Selection

The choice of electrode material is a critical factor influencing biosensor performance, affecting sensitivity, stability, biocompatibility, and cost. In this study, we systematically evaluated two common electrode materials, copper and gold, immersed in a medium with a relative permittivity ($\varepsilon_{r}$) approximating that of sweat ($\varepsilon_{r}\approx 80$). This simulation closely mimics the real-world operating conditions of the biosensor, where sweat serves as the target biofluid for biomarker analysis. The evaluation focused on the change in capacitance (ΔC) induced by the fluid, a key indicator of the sensor’s sensitivity to changes in the medium’s dielectric properties. Both copper and gold electrodes demonstrated a substantial increase in capacitance when immersed in a medium mimicking sweat ($\varepsilon_{r}\approx 80$) compared to air ($\varepsilon_{r}=1$), confirming their capability to detect changes in the dielectric environment. Specifically, copper electrodes exhibited a 76.19% increase in capacitance, while gold electrodes achieved a slightly higher increase of 78.46%.

Despite the marginal difference in sensitivity, copper was chosen as the electrode material for our biosensor due to its significantly lower cost. This decision aligns with our objective of developing an affordable device for widespread healthcare applications, where cost-effectiveness is paramount.

#### 3.3.2. Optimization of Electrode Geometry

To maximize the sensitivity of our biosensor to magnetic nanoparticles (MNPs) and, consequently, the biomarkers they bind, we systematically investigated the impact of electrode geometry on capacitive sensing performance. This optimization focused on two key parameters: electrode height (H) and microchannel width (W), with the aim of maximizing the capacitance (C), which is directly proportional to the electrode area (A = H × L, where L is the electrode length).

**Figure 8:**
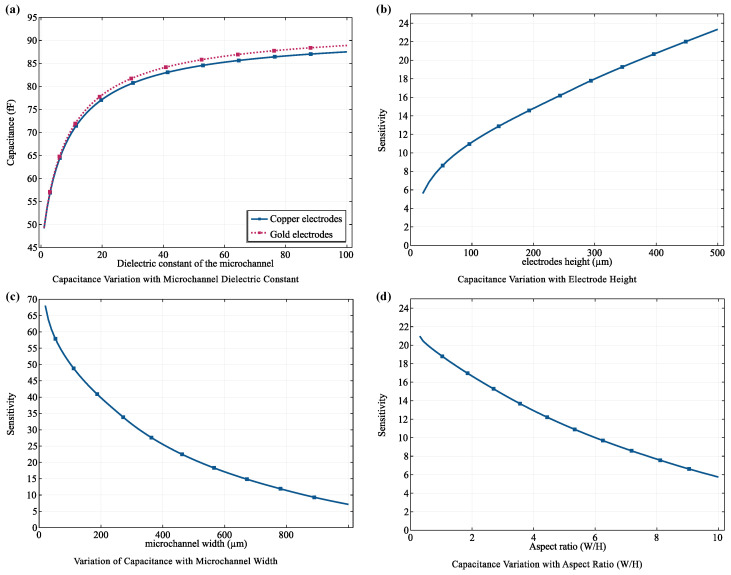
**Comprehensive Analysis of the Capacitive Detection System.** (**a**) Variation of capacitance as a function of dielectric constant for electrodes made from gold and copper, illustrating the electrostatic responsiveness of each material. (**b**) Sensitivity analysis of electrode height, showing how capacitance changes with increasing electrode dimensions. (**c**) Impact of electrode width on capacitance, demonstrating the inverse relationship between electrode width and capacitive sensitivity. (**d**) Analysis of the effect of aspect ratio ($\frac{W}{H}$) on system performance to identify optimal electrode dimensions for enhanced detection efficiency.

Figure 8b demonstrates a clear positive correlation between electrode height (H) and the sensor’s response. This aligns with the fundamental principle of capacitance, where increasing electrode area (proportional to height) leads to higher capacitance. This increased capacitance enhances the sensor’s sensitivity to changes in dielectric properties induced by the presence of MNPs in the detection zone.

Conversely, Figure 8c illustrates a negative correlation between microchannel width $\left(W_{\mathrm{ch}}\right)$ and capacitive sensitivity, which can be attributed to the influence of the inter-electrode gap (*d*). A wider microchannel increases *d*, reducing the overall capacitance and diminishing the sensor’s ability to detect subtle changes in response to MNPs. This inverse relationship underscores the importance of carefully selecting microchannel width to balance sensitivity with other design considerations, such as efficient MNP capture.

To achieve an optimal balance between sensitivity and practical considerations like microfabrication constraints and efficient MNP capture, we determined an aspect ratio $\left(\frac{W}{H}\right)$ of 1 to be ideal, as shown in Figure 8d. Setting the aspect ratio to 1 strikes a compromise, allowing for the design of electrodes with high sensitivity while maintaining practical feasibility for microfabrication processes and ensuring efficient MNP capture.

Based on these optimization results, we finalized the electrode dimensions to be 50 μm × 50 μm, with a detection zone length of 500 μm. This configuration ensures adequate coverage of the detection zone, allowing for optimal interaction between the magnetic field generated by the upper coil and the MNPs within the microchannel. The chosen length of 500 µm provides a sufficient electrode area for capturing MNPs and detecting changes in capacitance due to their presence, enhancing the biosensor’s ability to accurately detect and quantify biomarkers in diverse testing scenarios.

#### 3.3.3. Impact of MNP Properties and Concentration on Capacitive Detection

The relative permittivity ($\epsilon_{r}$) of commercially available magnetic nanoparticles (MNPs), such as ${\mathrm{Fe}}_{3}O_{4}$ and ${\mathrm{MnFe}}_{2}O_{4}$, varies significantly. To investigate the impact of this variation on the biosensor’s performance, we conducted comprehensive simulations across a range of $\epsilon_{r}$ values ranging from 10 to 40. Notably, these simulations were performed with MNPs alone, without the presence of target biomarkers, to isolate and directly evaluate the sensor’s response to changes in the dielectric properties of the medium solely induced by MNPs. This approach allowed us to confirm the fundamental operating principle of the DF-CS mechanism and its ability to detect changes in MNP concentration, which would, in a real-world scenario, be indicative of biomarker binding.

**Figure 9:**
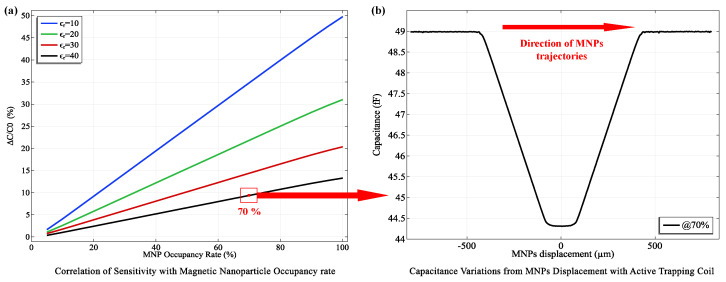
**Capacitive Sensing Performance of the Novel Biosensor.** (**a**) Graph illustrating the sensitivity of the biosensor as a function of magnetic nanoparticle (MNP) occupancy within the detection zone after trapping. (**b**) Capacitance response curve showing changes as MNPs are displaced within the detection zone, highlighting the biosensor’s dynamic response to variations in MNP concentration.

Figure 9a reveals a clear trend: sensitivity increases as $\epsilon_{r}$ decreases, particularly at higher MNP concentrations within the detection zone. This observation strongly supports the theoretical prediction that MNPs with lower $\epsilon_{r}$ values induce a more substantial shift in the effective dielectric constant of the detection zone, leading to greater capacitance changes detectable by the biosensor. Specifically, sensitivity improved from 9.01% at $\epsilon_{r}=40$ to 33.54% at $\epsilon_{r}=10$. Moreover, at a constant $\epsilon_{r}$ of 10, increasing the MNP concentration from 50% to 85% occupancy further amplified the sensitivity from 24.57% to 42.48%. These findings, in alignment with theoretical predictions from Equation (9), underscore the biosensor’s capability to detect and quantify biomarkers across a wide range of concentrations, including low-abundance targets critical for early disease detection.

To further assess the biosensor’s detection capabilities and dynamic response, we investigated how MNP displacement within the detection zone affects capacitance, as illustrated in Figure 9b. Initially, without MNPs, the capacitance remained stable at an average of 48.98fF (standard deviation: 0.036fF). Upon activating the R500 coil—selected for its high trapping efficiency and favorable power merit factor ($M_{p}$)—MNPs were introduced into the detection zone, reducing capacitance to 44.37fF (standard deviation: 0.095fF). This decrease of 4.60fF in mean capacitance provides robust evidence of the biosensor’s dynamic detection capabilities. Deactivation of the coil resulted in MNP release and a prompt return to baseline capacitance, highlighting the biosensor’s reliability, repeatability, and potential for continuous monitoring.

Overall, these simulations provide compelling evidence for the successful integration of the R500 microcoil with direct-field capacitive sensing and demonstrate the biosensor’s robust sensitivity and dynamic response to changes in MNP concentration. The optimized microcoil design, coupled with the direct-field approach, enables the precise capture, concentration, and quantification of MNP-tagged biomarkers, laying a strong foundation for future experimental validation and clinical translation of this promising biosensing platform.

## 4. Conclusions

In this study, we present the design and comprehensive 3D multiphysics simulation of a novel microfluidic immunosensor for real-time, non-invasive detection of pro-inflammatory biomarkers in human sweat. The integrated platform combines magnetofluidic manipulation for efficient biomarker capture and concentration with direct-field capacitive sensing (DF-CS) for label-free quantification. Notably, the sensor design prioritizes maximizing the interaction volume between a uniform electric field and the sample, a critical departure from traditional fringing-field capacitive sensors that can lead to non-uniform fields and reduced sensitivity. This direct-field approach ensures consistent and reliable biomarker detection.

Simulations demonstrate the sensor’s robust sensitivity to changes in dielectric medium properties, achieving a 42.48% change in capacitance at 85% magnetic nanoparticle (MNP) occupancy in the sensing region. This result, achieved without the actual biomarker present, validates the sensor’s core operating principle and its ability to translate MNP concentration into a quantifiable signal, which can then be correlated to biomarker levels. Optimized microcoil configurations and fluidic dynamics enable rapid biomarker-tagged MNP trapping and concentration, facilitating real-time monitoring, a critical feature for point-of-care diagnostics and personalized healthcare applications.

While this computational simulation study provides a promising foundation for a novel biosensing platform that addresses limitations of current diagnostic tools, potential challenges may arise during physical implementation. These challenges include maintaining uniform magnetic field strength across the microfluidic channel and mitigating potential interference from other sweat components in capacitive sensing. Addressing these challenges through calibration and optimization during microfabrication will be essential for future device optimization. Further research is needed to validate the findings of this computational simulation and assess the platform’s performance in real-world scenarios.

Future efforts will focus on experimental validation of these findings with clinically relevant biomarkers, such as tumor necrosis factor-alpha (TNF-α), and the development of a fully functional prototype for real-world assessment. By overcoming the limitations of current diagnostic tools, this non-invasive, continuous monitoring solution holds the potential for earlier detection of inflammatory conditions, personalized treatment interventions, and ultimately, improved patient outcomes.

## Figures and Tables

**Table 1:** Key Design Parameters for COMSOL Simulations.

Parameter Description	Symbol	Value Range
Current in the coil	$I_{\mathrm{coil}}$	100–700 mA
Electrode height	*H*	20–500 μm
Nanoparticle diameter	$D_{\mathrm{np}}$	10–60 nm
Coil-to-channel separation	$K_{\mathrm{coil}}$	5–100 μm
Microchannel width	$W_{\mathrm{ch}}$	50–1000 μm
Microchannel height	$H_{\mathrm{ch}}$	50 μm
Electrodes Voltage	*U*	5V
coil wire height	$H_{\mathrm{wire}}$	10 μm
coil wire width	$W_{\mathrm{wire}}$	10 μm
Fluid flow rate	*Q*	1 μL/min

**Table 2:** Key Material Constants for COMSOL Simulations.

Material Property	Symbol	Value
Copper Electrical conductivity	$\sigma_{Cu}$	59.6 MS/m ^(1)^
Gold Electrical conductivity	$\sigma_{Au}$	41 MS/m ^(1)^
Density of sweat	$\rho_{fluid}$	1000 kg/m^3^ ^(2)^
Dynamic viscosity of sweat	$\eta_{fluid}$	$10^{-3}$ Pa· s ^(3)^
PDMS Electrical conductivity	$\sigma_{PDMS}$	1.7 × $10^{-12}$ S/m ^(4)^
PDMS Relative permittivity	$\epsilon_{r,PDMS}$	2.7 ^(4)^
Permeability of magnetite nanoparticles	$\mu_{r,NP}$	5000 ^(5)^
Dielectric constant of MNPs	$\epsilon_{r,\mathrm{medium}}$	10–40 ^(6)^

^1^ A table of electrical conductivity of common materials. Available at: https://www.thoughtco.com/table-of-electrical-resistivity-conductivity-608499 (accessed on 22 July 2024). ^2^ Mark, J.E.: Polymer Data Handbook. Oxford University Press, 2009 [37]. ^3^ Robinson, S., Robinson, A.H.: Chemical composition of sweat. Physiological Reviews, 1954. ^4^ “PDMS Properties”, Massachusetts Institute of Technology, available at: https://www.mit.edu/~6.777/matprops/pdms.htm (accessed on 22 July 2024). ^5^ T. N. H. Nguyen et al., “Quantifying the complex permittivity and permeability of superparamagnetic iron-oxide nanoparticles”. Available: https://aip.scitation.org/doi/10.1063/1.4917489 (accessed on 22 July 2024). ^6^ S. Sahoo et al., “Enhanced Thermal Conductivity and Dielectric Properties of Iron Oxide”. Available: https://www.nature.com/articles/s41598-017-03273-z (accessed on 22 July 2024).

## Data Availability

The data supporting the findings of this study are available from the corresponding author upon reasonable request.
